# The organisation of physiotherapy for people with multiple sclerosis across Europe: a multicentre questionnaire survey

**DOI:** 10.1186/s12913-016-1750-6

**Published:** 2016-10-06

**Authors:** Kamila Rasova, Jenny Freeman, Patricia Martinkova, Marketa Pavlikova, Davide Cattaneo, Johanna Jonsdottir, Thomas Henze, Ilse Baert, Paul Van Asch, Carme Santoyo, Tori Smedal, Antonie Giæver Beiske, Małgorzata Stachowiak, Mariusz Kovalewski, Una Nedeljkovic, Daphne Bakalidou, José Manuel Alves Guerreiro, Ylva Nilsagård, Erieta Nikolikj Dimitrova, Mario Habek, Kadriye Armutlu, Cécile Donzé, Elaine Ross, Ana Maria Ilie, Andrej Martić, Anders Romberg, Peter Feys

**Affiliations:** 1 Department of rehabilitation, Third Faculty of Medicine, Charles University in Prague, Ruska 87, Prague 10, 100 00 Czech Republic; 2 Faculty of Health and Human Science, Plymouth University, Playmouth, PL6 8BH England; 3 Institute of Computer Science, Academy of Sciences of the Czech Republic, Pod Vodarenskou vezi 2, Prague 8, 182 07 Czech Republic; 4 Third Faculty of Medicine, Charles University in Prague, Ruska 87, Prague 10, 100 00 Czech Republic; 5 Don Gnocchi Foundation, Larice Lab, Santa Maria Nascente, Via Capecelatro 66, 20148 Milan, Italy; 6 Neurorehabilitation at the Don Gnocchi Foundation, Larice Lab, Santa Maria Nascente, Via Capecelatro 66, 20148 Milan, Italy; 7 PASSAUER WOLF Reha-Zentrum Nittenau, Rehabilitations klinik für Neurologie-Geriatrie-Urologie, Eichendorffstr. 21, D-93149 Nittenau, Germany; 8 Hasselt University, Campus Diepenbeek, REVAL Rehabilitation Research Institute (BIOMED), Agoralaan building A, B-3590 Diepenbeek, Belgium; 9 Fit Up, Fitness- and Physiotherapy Center, Mechelsesteen weg 192a, 2550 Kontich, Belgium; 10 Cemcat, Neurorehabilitation Unit, Passeig de la Vall d’Hebron, 119-129, 08035 Barcelona, Spain; 11 Department of Neurology, Norwegian Multiple Sclerosis Competence Centre, Haukeland University Hospital, P.O. Box 1400, 5021 Bergen, Norway; 12 Department of Physiotherapy, Haukeland University Hospital, P.O. Box 1400, 5021 Bergen, Norway; 13 MS-Senteret Hakadal AS, Blomsterbakken 33, 1487 Hakadal, Norway; 14 MS Rehabilitation Centre, Szpitalna 5, 78-449 Borne Sulinowo, Poland; 15 Clinic for physical medicine and rehabilitation, Clinical Center of Serbia, Pasterova 2, 11000 Belgrade, Serbia; 16 Technological Educational Institute of Athens, 24, Mitrodorou street, Ak. Pratonos, 10441 Athens, Greece; 17 School of Health Sciences, Health Research Unit, Polytechnic Institute of Leiria, Campus 2 - Morro do Lena - Alto do Vieiro, 2411-901 Leiria, Portugal; 18 Faculty of Medicine and Health, Örebro University Region, SE- 701 82 Örebro, Sweden; 19 Institute of Physical Medicine and Rehabilitation, Faculty of Medicine, “Ss Cyril and Methodius” University, Elisie Popovski 28, 1000 Skopje, Macedonia, Republic of The former Yugoslav; 20 Referral Center for Demyelinating Diseases of the Central Nervous System University Department of Neurology Zagreb School of Medicine and University Hospital Center, Kispaticeva 12, HR-10000 Zagreb, Croatia; 21 Physical Therapy and Rehabilitation departmant of Health Science Faculty, Hacettepe University, Ankara, Turkey; 22 Groupe Hospitalier de l’institut Catholique de Lille, Department of physical Medicine and Rehabilitation hospital Saint Philibert, Faculté Libre de Médecine, Univ Nord de France, F-59000 Lille, France; 23 St. James’s Hospital, St. James’s street, Dublin 8, Ireland; 24 “Elias” University Emergency Hospital, 17 Marasti Bulevard, Bucharest, 01146 Romania; 25 Divison of Neurology, Neurorehabilitation unit, University Medical Centre Ljubljana, Zaloška 2, 1000 Ljubljana, Slovenia; 26 Masku Neurological Rehabilitation Centre, Physiotherapy, Vaihemäentie 10, PO Box 15, 21251 Masku, Finland

## Abstract

**Background:**

Understanding the organisational set-up of physiotherapy services across different countries is increasingly important as clinicians around the world use evidence to improve their practice. This also has to be taken into consideration when multi-centre international clinical trials are conducted. This survey aimed to systematically describe organisational aspects of physiotherapy services for people with multiple sclerosis (MS) across Europe.

**Methods:**

Representatives from 72 rehabilitation facilities within 23 European countries completed an online web-based questionnaire survey between 2013 and 2014. Countries were categorised according to four European regions (defined by United Nations Statistics). Similarities and differences between regions were examined.

**Results:**

Most participating centres specialized in rehabilitation (82 %) and neurology (60 %), with only 38 % specialising in MS. Of these, the Western based Specialist MS centres were predominately based on outpatient services (median MS inpatient ratio 0.14), whilst the Eastern based European services were mostly inpatient in nature (median MS inpatient ratio 0.5). In almost all participating countries, medical doctors - specialists in neurology (60 %) and in rehabilitation (64 %) - were responsible for referral to/prescription of physiotherapy. The most frequent reason for referral to/prescription of physiotherapy was the worsening of symptoms (78 % of centres). Physiotherapists were the most common members of the rehabilitation team; comprising 49 % of the team in Eastern countries compared to approximately 30 % in the rest of Europe. Teamwork was commonly adopted; 86 % of centres based in Western countries utilised the interdisciplinary model, whilst the multidisciplinary model was utilised in Eastern based countries (*p* = 0.046).

**Conclusion:**

This survey is the first to provide data about organisational aspects of physiotherapy for people with MS across Europe. Overall, care in key organisational aspects of service provision is broadly similar across regions, although some variations, for example the models of teamwork utilised, are apparent. Organisational framework specifics should be considered anytime a multi-centre study is conducted and results from such studies are applied.

## Background

It is suggested that organisational aspects of physiotherapy (PT), such as the services and types of programs offered to patients, selection criteria for admission into rehabilitation, or the intensity of therapy influence outcomes of therapeutic interventions [1–5]. However, there are no international comparative reports mapping similarities or differences in the organisation of PT in multiple sclerosis (MS), and the quality of description in most studies about these aspects is remarkably poor [6]. This type of information is important as evidence is increasingly being used by clinicians from across the world to improve their practice [7]. In addition, with the recognition that larger sample sizes are needed to provide a robust scientific basis for the evidence generated, it is becoming more common for multi-centre clinical trials across different countries to be designed and implemented. An enhanced understanding of the organisational context in which PT interventions are provided will enhance our understanding as to whether the results from these multi-centre trials are generalizable across different countries.

## Methods

### Description of the project

The overall project “Content of physiotherapy in multiple sclerosis – questionnaire study, COPHYREQUEST” consisted of two phases. In the first phase, carried out between 2010 and 2012, the survey questionnaires were developed and a list of potential participants was prepared. In the second phase, carried out between 2013 and 2014, the two surveys were implemented. The first questionnaire survey aimed to systematically describe organisational aspects of PT services for people with multiple sclerosis (MS) across Europe (results are described in this article). For the purposes of this study, PT was defined as a health discipline that aims to develop, maintain and restore maximum movement and functional ability throughout the lifespan [8]. The second questionnaire survey (to be reported elsewhere) focused on describing the physiotherapists’ level of awareness and knowledge about different assessment and therapeutic PT approaches used within this patient group.

#### Research design

A descriptive, cross-sectional survey, using convenience sampling.

### The survey questionnaire

A literature search highlighted no relevant pre-existing survey questionnaire to describe organisational aspects of PT services. Consequently a questionnaire was developed, in line with established design principles [9], as described below.

The core group within the Mobility Special Interest Group (SIG) of the Rehabilitation in Multiple Sclerosis European network of best practice and research in MS rehabilitation (RIMS, www.euRIMS.org) were involved in the development of this semi-structured questionnaire. The lead author developed an initial draft of the questionnaire. It was piloted with 56 health professionals (medical doctors and physiotherapists) attending a RIMS workshop in Prague, 2010. All workshop participants were involved in the management of mobility of people with MS. Subsequent iterations were undertaken by the core group members (*n* = 12) via e-mail until a version was developed which was considered suitable for further piloting at a second RIMS workshop one year later in Barcelona (*n* = 46). Agreement on the final questionnaire items and wording followed after one more round of e-mail communication. The questionnaire used both closed and open-ended questions; the latter were designed to elicit descriptions and opinions. The internet version of the questionnaire was prepared based on guidelines described by Cooper et al., 2006 [10].

The Questionnaire comprised 30 questions, covering a range of topics including: specialization of the centre, number of MS inpatients and outpatients seen per year, number and type of professionals in the rehabilitation team treating MS, the professions who referred patients for PT and their reasons for doing so, the format of PT sessions that the centre offered both for inpatients and outpatients (individual, group, autonomous), and a description of the typical therapeutic session (length, frequency and number of sessions). Respondents were encouraged to consult with their colleagues regarding their responses and to draw on written materials and records from their workplace (e.g. patient case notes).

#### Recruiting process

The databases of RIMS, European Multiple Sclerosis Platform, European Society of Physical and Rehabilitation Medicine, World Federation for NeuroRehabilitation, and professional networks LinkedIn and ResearchGate were searched to identify key individuals working in the field of MS rehabilitation who were able to describe service provision within their country, and identify potential respondents for the questionnaire survey. Individuals from 45 European countries were identified and contacted. Of these, representatives from 28 countries confirmed their participation and, according to their best knowledge and experience, identified centres in their country that fulfilled the inclusion criteria (detailed below). A list of all candidate/eligible centres was compiled (202 centres in all). Information about the survey was also advertised at relevant MS conferences and international meetings. Representatives of centres fulfilling the inclusion criteria were informed about the survey and asked to participate. To enhance validity of the results, they were assured that the names of centres would not be published in order to eliminate any potential fear of comparison between centres. Country representatives contacted eligible participating centres regularly to optimise the survey response rate.

### Inclusion criteria

Centres were eligible for inclusion if they provided facilities/workplaces where people with MS could engage in PT. Respondents were eligible to complete the survey if they had relevant expertise and access to information about key organizational aspects of their centre; for example, if they were the head of the rehabilitation facility.

#### Data analyses

Countries were divided into four European regions defined by United Nations Statistics Department [11] (Table 1). Data were analysed for the whole sample, as well as separately for each of the four regions. They are presented in tables and figures as medians and interquartile range (IQR) or as means of proportions where appropriate. Differences between regions were assessed through Kruskal-Wallis, Fisher exact test or χ^2^-test where appropriate. To fulfil minimum cell counts assumption for χ^2^-test some categories were joined together for some parameters. For the profession load distribution χ^2^-test (Question 9), the following professions were pooled: sport instructors, occupational therapists and speech/swallowing therapists into “specialised physical care” category, social workers and psychologists into “psychosocial care”. A sensitivity analysis joining the “other” category to either the “physical” or “psychosocial” category was performed. The level of statistical significance was set to 0.05. Statistical language and environment R, version 3.1.2, was used throughout the analyses.

**Table 1:** Survey participants – size of the centers (in patients per year) their multiple sclerosis (MS) specialization and proportion of MS patients using outpatient compared to inpatient services

Region	Countries [N]	Centers asked for participation [N]	Centers participating [N]	Response rate [%]	MS inpatients [median (IQR)]	MS outpatients [median (IQR)]	Total inpatients [median (IQR)]	Total outpatients [median (IQR)]	MS ratio [median (IQR)]	MS inpatient ratio [median (IQR)]
Europe	23	193	72	37	28 (99)	30 (106)	300 (998)	375 (1906)	0.11 (0.47)	0.33 (0.88)
East	3	37	9	24	12 (20)	10 (0)	450 (610)	750 (800)	0.03 (0.06)	0.50 (0.98)
North	7	64	23	36	35 (90)	35 (120)	220 (952)	155 (2124)	0.16 (0.41)	0.42 (0.98)
South	9	70	28	40	20 (70)	48 (124)	300 (976)	500 (1908)	0.11 (0.76)	0.30 (0.64)
West	4	22	12	55	65 (132)	30 (112)	300 (990)	500 (1162)	0.10 (0.36)	0.14 (0.86)
Region	Country	Centers asked for participation [N]	Centers participating [N]	Response rate [%]	MS inpatients [median (IQR)]	MS outpatients [median (IQR)]	Total inpatients [median (IQR)]	Total outpatients [median (IQR)]	MS ratio [median (IQR)]	MS inpatient ratio [median (IQR)]
East	Czech Republic	20	7	35	10 (14)	10 (5)	400 (1265)	852 (725)	0.01 (0.03)	0.33 (0.75)
	Poland	13	1	8	560 (0)	10 (0)	610 (0)	750 (0)	0.42 (0.00)	0.98 (0.00)
	Romania	4	1	25	200 (0)	150 (0)	450 (0)	200 (0)	0.54 (0.00)	0.57 (0.00)
North	Denmark	2	1	50	900 (0)	0 (0)	900 (0)	0 (0)	1.00 (0.00)	1.00 (0.00)
	Estonia	3	3	100	60 (105)	250 (248)	1100 (750)	6000 (10625)	0.02 (0.05)	0.33 (0.35)
	Finland	5	1	20	730 (0)	67 (0)	1300 (0)	71 (0)	0.58 (0.00)	0.92 (0.00)
	Ireland	10	4	40	0 (6)	38 (28)	0 (62)	228 (286)	0.14 (0.07)	n.a.
	Norway	16	8	50	54 (82)	0 (22)	285 (416)	65 (991)	0.23 (0.45)	1.00 (0.41)
	Sweden	12	5	42	24 (95)	140 (150)	95 (95)	900 (5345)	0.17 (0.43)	0.05 (1.00)
	United Kingdom	16	1	6	35 (0)	80 (0)	120 (0)	160 (0)	0.41 (0.00)	0.30 (0.00)
South	Croatia	6	3	50	185 (72)	28 (498)	1100 (1225)	2000 (2195)	0.04 (0.23)	0.87 (0.38)
	FYROM	6	2	33	16 (4)	12 (8)	1020 (980)	4045 (3955)	0.06 (0.05)	0.64 (0.14)
	Greece	6	2	33	65 (15)	165 (135)	775 (425)	1150 (50)	0.14 (0.11)	0.42 (0.21)
	Italy	4	4	100	40 (22)	36 (497)	100 (453)	93 (4975)	0.10 (0.22)	0.50 (0.24)
	Portugal	16	5	31	0 (1)	15 (43)	54 (456)	350 (1720)	0.02 (0.05)	0.00 (0.01)
	Serbia	7	4	57	90 (160)	50 (15)	885 (2048)	4500 (7125)	0.01 (0.05)	0.71 (0.20)
	Slovenia	5	1	20	100 (0)	1800 (0)	150 (0)	2000 (0)	0.88 (0.00)	0.05 (0.00)
	Spain	20	6	30	0 (2)	49 (76)	0 (5)	54 (102)	0.85 (0.10)	0. (0.04)
	Turkey	7	1	14	100 (0)	200 (0)	1000 (0)	300 (0)	0.23 (0.00)	0.33 (0.00)
West	Belgium	7	5	71	70 (55)	50 (133)	430 (880)	300 (475)	0.19 (0.9)	0.45 (0.78)
	France	8	1	13						
	Germany	6	2	33	475 (325)	5 (5)	1275 (25)	75 (75)	0.38 (0.26)	0.97 (0.03)
	Netherlands	4	4	100	2 (6)	65 (120)	100 (225)	1250 (2750)	0.04 (0.02)	0.02 (0.06)

*N* number, *MS* multiple sclerosis, *IQR* interquartile range

MS inpatients = median (IQR) number of MS patients per year the participating centers take care of on inpatient basis in the given region/country

MS outpatients = median (IQR) number of MS patients per year the participating centers take care of on outpatient basis in the given region/country

Total inpatients = median (IQR) number of all type patients per year the participating centers take care of on inpatient basis in the given region/country

Total outpatients = median (IQR) number of all type patients per year the participating centers take care of on outpatient basis in the given region/country

MS outpatients = median (IQR) number of MS patients the participating centers in the given region/country take care of on outpatient basis

MS ratio: median (IQR) accros region/country of ratio of MS patients to total number of patients calculated for each center separately (range from 0 – center not specialized in MS to 1 - center fully specialized in MS). No statistically significant difference between regions (Kruskal-Wallis test; *p* = 0.166)

MS inpatient ratio: median (IQR) accros region/country of ratio of patients in inpatient care to total care offered to MS patients (range from 0 – center provides outpatient care only to 1 – center provides outpatient care only). No statistically significant difference between regions (Kruskal-Wallis test; *p* = 0.541)

## Results

From a potential 45 European countries, 28 country coordinators agreed to cooperate in the study. Of those, 23 coordinators recruited 72 centre representatives to complete the survey (Table 1; those failing to recruit are not published for reasons of confidentiality). Region wise, Eastern Europe was represented by three countries out of a possible 10 (Czech Republic, Poland, Romania), Northern Europe by seven out of a possible 10 (Denmark, Estonia, Finland, Ireland, Norway, Sweden, United Kingdom), Southern Europe by nine out of a possible 16 (Croatia, Former Yugoslav Republic of Macedonia - FYROM, Greece, Italy, Portugal, Serbia, Slovenia, Spain, Turkey) and Western Europe by four out of a possible nine countries (Belgium, France, Germany, Netherlands). Eastern Europe is hence the least represented region in the survey, both with regard to the participating countries and the number of centres that responded to the country coordinators invitation to participate.

Centre representatives that completed the survey were either leaders of the centres/rehabilitation departments (*n* = 27, 38 %), heads of PT (*n* = 19, 26 %) or specialists in PT (for example, physiotherapists, medical doctors, sports instructors, *n* = 26, 36 %).

The overall response rate for the survey questions was very good. It should be noted that not all questions were relevant to all respondents. For example, in centres exclusively providing outpatient care, the questions relating to inpatient care were not relevant and therefore not answered. Taking this into consideration, on most questions only 0–2 answers were missing. Four respondents (6 %) of 66 centres providing outpatient care failed to provide details about their outpatient PT sessions.

### Size, specialization and proportion of MS patients using outpatient compared to inpatient services (Table 1)

*Question 6–9: Respondents were asked to estimate the number of MS in/outpatients and all-diagnoses in/outpatients in their centre or PT department. They were asked about the neurology/rehabilitation/MS specialisation/other character of their department.*

Size of participating centers expressed in numbers of MS inpatients and outpatients per year varied extensively, from centers with only tens of MS patients per year to centers with thousands of patients per year. Specialization of centers, expressed as the MS ratio (ratio of total MS patients to total number of patients seen on either an inpatient or outpatient basis) ranged from almost zero to 100 %. The median MS ratio (Table 1) demonstrates that, in all European regions, rehabilitation is mainly offered in centres that are not specialised in MS. Participating centres were mostly specialized in rehabilitation (82 %) and neurology (60 %), with fewer facilities specializing in MS (38 %). In Eastern Europe only one of nine participating centres (11 %) reported specialization in MS rehabilitation, in contrast to centres from across the rest of Europe where approximately 40 % provided MS specialist rehabilitation facilities (Table 2).

**Table 2:** Specialisation of participating centres (percentage of centres offering the given specialisation

Specialization	Europe [%] (*n* = 72)	East [%] (*n* = 9)	North [%] (*n* = 23)	South [%] (*n* = 28)	West [%] (*n* = 12)	Fisher test *p*-value*
Neurology	59.7	22.2	73.9	64.3	50.0	0.0534
Rehabilitation	81.9	88.9	69.6	82.1	100.0	0.1644
MS	37.5	11.1	43.5	39.3	41.7	0.3913
Other	18.1	22.2	21.7	14.3	16.7	0.8909

The percentages do not sum up to 100 % as some centres checked more than one specialization

*p-values of Fisher exact test for each specialization. When Bonferroni correction is applied for 4 comparisons (level of significance 0.0125) the differences are not statistically significant

The MS inpatient ratio (ratio of total MS inpatients to total number of MS patients of either inpatient or outpatient basis, Table 1) also varied among European countries. Some centres only provide outpatient services, whilst others exclusively offer inpatient care. At a European regional level, participating Western centres mostly provide outpatient services (median MS inpatient ratio 0.14), whilst participating services in the East are mostly inpatient services (median MS inpatient ratio 0.5), although overall, the differences were not statistically significant (*p* = 0.166).

### Type of teamwork

*Question 10: Respondents were asked to choose which description best fits their centre. A multidisciplinary team was defined as “specialists who work in parallel towards addressing problems related to their profession”. An interdisciplinary team was defined as “specialists working as a group to achieve a common goal that is explicitly agreed upon”. Other choices included “individual PT practice” and “other (please specify)”.*

**Fig. 1:**
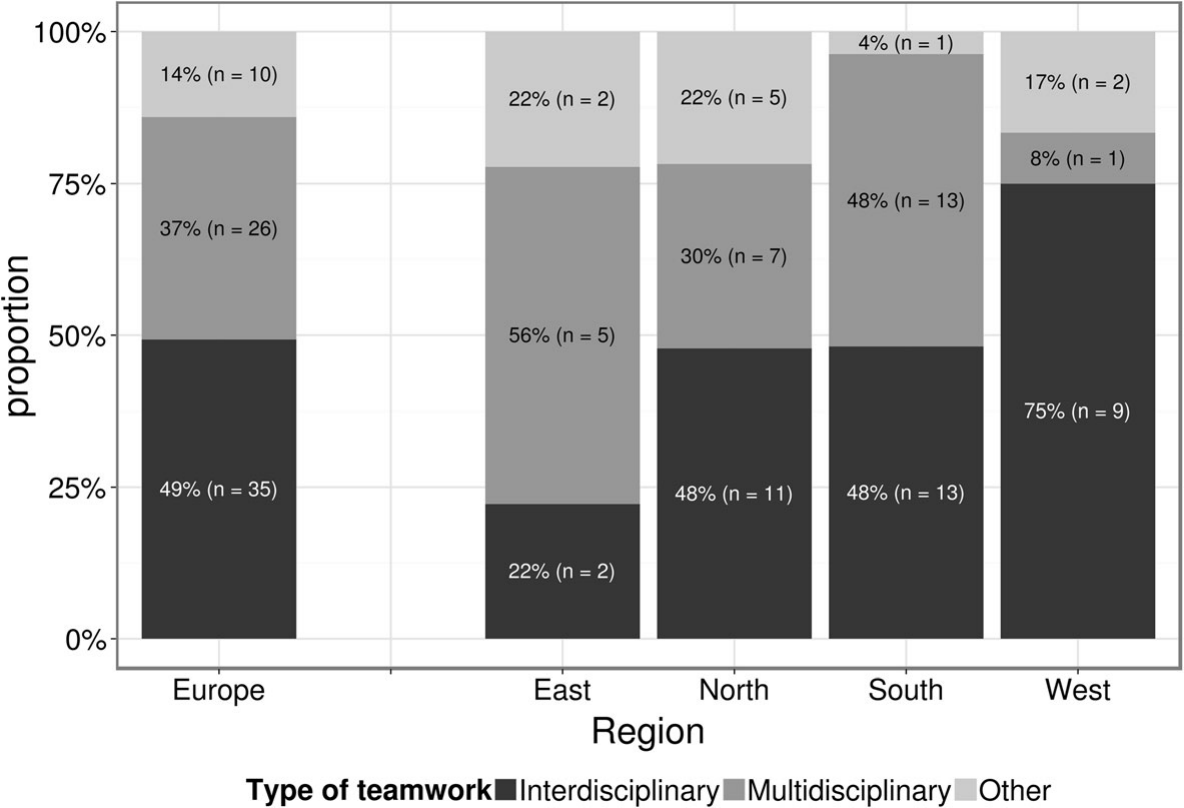
Type of teamwork across Europeans regions. Graph shows proportion of answers by 72 respondents together with counts in parentheses. Multidisciplinary team was defined as “specialists work in parallel towards addressing problems related to their profession”. Interdisciplinary team was defined as “specialists working as a group to achieve a common goal that is explicitly agreed upon”. Type of teamwork differs across the regions (Fisher exact test; *p* = 0.046)

Most participating centres (*n* = 61, 86 %) reported using a teamwork approach. Almost half (49 %) used the interdisciplinary model, whilst 37 % used a multidisciplinary teamwork approach. Results suggest that in the Western countries the interdisciplinary teamwork prevails, where specialists are working together for the same goal. In contrast in Eastern countries the multidisciplinary model, where efforts of different team members are parallel and discipline-oriented, is more frequent (Fig. 1, the differences were statistically significant, *p* = 0.046).

### Number and distribution of professionals

*Question 11: Respondents were asked to specify the number of employees of listed professions in their workplace treating MS patients. Half-time employees counted as 0.5 etc.*

*Question 12: Who prescribes/recommends physiotherapy in patients with MS in your workplace?*

**Fig. 2:**
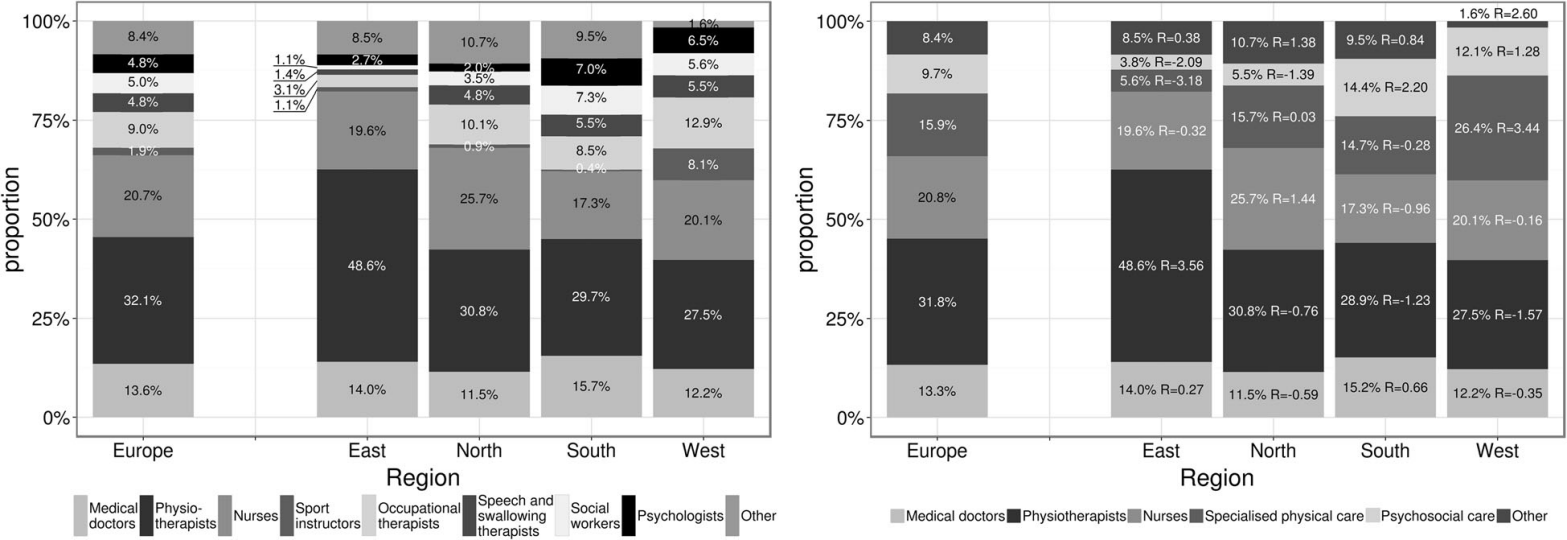
Distribution of professionals and groups of professionals in the team, for different European regions. Mean proportion of various professions in the team across regions and Europe in given in the graphs. For the χ^2^-test the professions were pooled as follows: sport instructors, occupational therapists and speech/swallowing therapists into “specialised physical care” category, social workers and psychologists into “psychosocial care”. There was a statistically significant difference between regions in the distribution of professionals in teams (*p* < 0.001). Standardised residuals between observed and expected proportions are given in the second graph. Sensitivity analysis with joining “other” category to either “physical” or “psychosocial” category was performed and confirmed the statistically significant difference between regions (*p* = 0.015 and *p* = 0.002 respectively)

Based on respondent answers, the distribution of professional load for each centre was computed. The mean distribution per region and in the whole respondent set is shown in Fig. 2.

In all regions physiotherapists are the most common members of the rehabilitation team (European mean 32 %). This is most apparent in Eastern countries (49 %) compared to the rest of Europe (approximately 30 %). Eastern countries also have the largest proportion (82 %) of medically educated therapists (MD, physiotherapists, nurses) compared to a European mean of 66 %. Many specialists such as occupational therapists, sport instructors or speech and swallowing therapists were more frequently present in Western countries; there were fewer of these specialists in Northern and Southern countries, and these were rarely present in Eastern countries. Psychosocial care professionals (social workers, psychologists) were rarely reported as team members within centres in Eastern countries but were often present in centres in Southern and Western countries. The differences between regions were statistically significant (*p* < 0.001) (Fig. 2).

### Who referred to/prescribed physiotherapy services

*Question 13: Who prescribes/recommends physiotherapy in your workplace?*

In almost all participating countries, medical doctors - specialists in neurology (60 %) and in rehabilitation (64 %) - were reported as being responsible for referral to/prescription of PT (Table 3). Sometimes people with MS were reported to be able to self refer to a physiotherapist, most typically in Northern countries (as specified by respondents when they checked the “other” option).

**Table 3:** Health care professional who referred to/prescribed physiotherapy services

Type of professional	Europe [%] (*n* = 72)	East [%] (*n* = 9)	North [%] (*n* = 23)	South [%] (*n* = 28)	West [%] (*n* = 12)	Fisher test *p*-value*
MD Neurologist	59.7	66.7	73.9	39.3	75.0	0.0482
MD Rehabilitation Specialist	63.9	77.8	47.8	64.3	83.3	0.1753
MD General Practitioner	20.8	22.2	39.1	3.6	25.0	0.0088**
Physiotherapist	19.4	11.1	43.5	3.6	16.7	0.0028**
Other	13.9	0.0	30.4	7.1	8.3	0.0710

The percentages do not sum up to 100 % as some respondents checked more than one option. “Other” option was often specified as patient self-referral

*p-values of Fisher exact test for each referring profession. When Bonferroni correction is applied for 5 comparisons (level of significance 0.01) the differences between regions in the prescriptive possibility of General Practicioner and Physiotherapist are statistically significant

**Statistically significant result at 5 % significance level

### Reasons why people with MS are referred/prescribed physiotherapy

*Question 14: Respondents were asked to tick reasons for physiotherapy prescription in the workplace.*

The most frequent reason for referral to/prescription of PT was for: the worsening of symptoms (78 % of centres, Table 4), preventive care (71 %), the management of an acute exacerbation (58 %), palliative care (40 %), and for psychosocial issues (38 %). Our data suggest that, in general, centres in Western countries prescribe PT for a greater variety of reasons than other countries (Table 4, last row) and that in Eastern countries PT is rarely prescribed for preventive or palliative care, although the differences were not statistically significant (*p* = 0.172 for variety of reasons, *p* = 0.554 for preventive care and *p* = 0.019 for palliative care respectively; multiple comparisons correction applies). To get better insight into the latter, we pooled “hard” reasons into one group (“Diagnosis”, “Acute exacerbation” and “Worsening of symptoms”) and “soft” reasons into another (“Preventive”, “Palliative” and “Psychological care”). Although the data suggest numerical differences between Eastern and other regions, we cannot say that the difference is statistically significant in soft reasons for physiotherapy (*p* = 0.2552).

**Table 4:** Reasons for referral/prescription of physiotherapy services

Reason for prescription	Europe [%] (*n* = 72)	East [%] (*n* = 9)	North [%] (*n* = 23)	South [%] (*n* = 28)	West [%] (*n* = 12)	Fisher test *p*-value*
Diagnosis as such	63.9	55.6	78.3	50.0	75.0	0.1566
Acute exacerbation	58.3	55.6	56.5	60.7	58.3	0.9849
Worsening of symptomes	77.8	88.9	65.2	82.1	83.3	0.4506
Preventive care	70.8	55.6	73.9	67.9	83.3	0.5538
Paliative care	40.3	0.0	34.8	50.0	58.3	0.0186
Psychological issues	37.5	22.2	26.1	39.3	66.7	0.1037
Other	8.3	0.0	8.7	10.7	8.3	0.9339
Hard health reasons	95.8	88.9	95.7	100.0	91.7	0.2190
Soft health reasons	77.8	55.6	73.9	82.1	91.7	0.2552
Number of reasons [median]	4	3.0	4	3.5	4.5	0.1720^a^

The percentages do not sum up to 100 % as some centres checked more than one option

Hard health reasons group consists of “Diagnosis as such”, “Acute exacerbation” and “Worsening of symptomes”. Soft health reasons group consists of “Preventive, paliative and psychological care” reasons

*p-values of Fisher exact test for each reason. When Bonferroni correction is applied for 7 comparisons (level of significance 0.007) there are no statistically significant differences between regions

^a^Kruskall-Wallis test for number of reasons given by center representatives

### Type of treatment

*Question 15 and 25: What kind of physiotherapy does your workplace offer to people with MS as part of the inpatient (Question 15)/outpatient (Question 25) program?*

Of the participating centres, 51 (71 %, Table 5) provided inpatient care, 62 (86 %) provided outpatient care, and 41 (57 %) provided both. Based on their responses it is apparent that people with MS throughout Europe mainly receive treatment on an individual basis (96 % of centres providing an inpatient service, and 94 % of centres providing an outpatient service). Group therapy is less common (45 % of the centres offer it on an inpatient basis and 40 % on an outpatient basis); although it is much more common in the West and North regions compared to the South and East regions. Autonomous therapy – led by an independent, self-determined professional as defined by American Physical Therapy Association [12] – is offered by only 14 % of centres on an inpatient basis and by 10 % of centres to outpatients.

**Table 5:** Type of treatment

Type of therapy provided		Europe [%] (*n* = 72)	East [%] (*n* = 9)	North [%] (*n* = 23)	South [%] (*n* = 28)	West [%] (*n* = 12)	Fisher test *p*-value
Centers providing inpatient therapy		51 (71 %)	6 (66 %)	17 (74 %)	20 (71 %)	8 (67 %)	0.9468
Of those offer	Inpatient individual	49 (96 %)	6 (100 %)	16 (94 %)	20 (100 %)	7 (88 %)	
	Inpatient group	23 (45 %)	3 (50 %)	9 (53 %)	6 (30 %)	5 (63 %)	
	Inpatient autonomous	7 (14 %)	1 (17 %)	4 (24 %)	2 (10 %)	0 (0 %)	
	Inpatient other	2 (4 %)	0 (0 %)	1 (6 %)	0 (0 %)	1 (13 %)	
Centers providing outpatient therapy		62 (86 %)	7 (78 %)	17 (74 %)	28 (100 %)	10 (83 %)	0.0130
Of those offer	Outpatient individual	58 (94 %)	7 (100 %)	16 (94 %)	25 (89 %)	10 (100)	
	Outpatient group	25 (40 %)	0 (0 %)	9 (53 %)	9 (32 %)	7 (70 %)	
	Outpatient autonomous	6 (10 %)	0 (0 %)	2 (12 %)	4 (14 %)	0 (0 %)	
	Outpatient other	6 (10 %)	0 (0 %)	3 (18 %)	2 (7 %)	1 (10 %)	
Centers providing individual therapy on any basis		71 (99 %)	9 (100 %)	23 (100 %)	28 (100 %)	11 (92 %)	0.2917
Centers providing group therapy on any basis		37 (52 %)	3 (33 %)	15 (65 %)	9 (32 %)	10 (83 %)	0.0071
Centers providing both in- and outpatient therapy		41 (57 %)	4 (44 %)	11 (48 %)	20 (71 %)	6 (50 %)	0.2654

The table provides number and percentage of centres providing inpatient and outpatient therapy across regions and in total (row 1 and 5). Then it provides number and percentage of centers providing individual/group/autonomous/other calculated with respect to centres offering the inpatient/outpatient care in the defined area

### Typical therapy

*Questions 16 to 24 and 26 to 34: Describe standard therapeutic program for individual/group/autonomous therapy in an inpatient/outpatient setting: total number of weeks per year, average number of sessions per week, average duration of a session in minutes.*

*Question 35: Does the number of weeks, number of sessions per week and/or duration of therapy vary with disease severity?*

Detailed information about standard therapy (number of weeks per year, number of sessions and duration of sessions) is shown in Table 6. All 51 of the centres providing inpatient care and 62 of the centres providing outpatient care gave details about the service organisation, but there appeared to be a misunderstanding that the frequency in weeks should refer to one patient and not to staff workload. Therefore, data from a smaller proportion of centres (particularly those providing outpatient services) were available for computation of dosage.

**Table 6:** Standards of physiotherapy provision in inpatient care (51 centers) and outpatient care (62 centers)

Inpatient individual	Number of centers providing the type of care	Weeks/year median (IQR)	Sessions/week median (IQR)	Minutes/sessions median (IQR)	Number of centers providing the data to compute the dosage	Dosage in hours/year median (IQR)
Europe	49	4 (8)	5 (1.2)	45 (30)	38 (78 %)	12.6 (27.5)
East	6	4 (1)	5.5 (4.8)	52 (15)	6 (100 %)	32 (32.1)
North	16	4 (24)	4 (1)	45 (20)	11 (69 %)	10 (1.8)
South	20	4 (16)	5 (0.2)	45 (15)	15 (75 %)	20 (29.4)
West	7	6 (5)	5 (4.5)	30 (5)	6 (86 %)	24.5 (19.4)
Inpatient group						
Europe	23	4 (7)	5 (5)	45 (16)	19 (83 %)	24 (30.4)
East	3	4 (1)	6 (3.5)	45 (15)	3 (100 %)	24 (19.5)
North	9	4 (4)	5 (5.2)	45 (5)	7 (78 %)	18 (16.4)
South	6	4 (13)	5 (2.2)	52 (60)	5 (83 %)	36 (28.8)
West	5	8 (21)	10 (9)	40 (15)	4 (80 %)	51.5 (67)
Inpatient autonomous						
Europe	7	4 (22)	5 (1.5)	30 (5)	5 (71 %)	6.0 (1.5)
Outpatient individual						
Europe	58	12 (36)	2 (2)	45 (20)	42 (72 %)	15 (16)
East	7	8 (14)	2 (2.5)	45 (22)	6 (86 %)	9.8 (5.6)
North	16	12 (30)	2 (3)	52 (15)	11 (69 %)	9 (14)
South	25	10 (41)	2 (3)	45 (15)	18 (72 %)	23.2 (21.1)
West	10	16 (33)	2 (0)	35 (15)	7 (70 %)	13.5 (11)
Outpatient group						
Europe	25	10 (44)	2 (2)	60 (15)	8 (32 %)	40 (30.2)
East	0				0	
North	9	10 (11)	2 (1)	60 (15)	2 (22 %)	71 (29)
South	9	20 (46)	3 (3)	45 (15)	5 (56 %)	30 (25)
West	7	10 (43)	2 (1.5)	60 (18)	1 (14 %)	150 (0)
Outpatient autonomous						
Europe	6	45 (37)	5.5 (1.8)	38 (26)	3 (50 %)	15 (54)

While all 51/62 centers providing inpatient/outpatient care gave details about frequency and timing of care, some misunderstood that the frequency in weeks should reffer to one patient and not to staff workload. Therefore, data from smaller proportion of centers could have been used for dosage computation

Becuse of small number of centers prividing autonomous therapy, data are not broken down by region. Even so, estimates of session frequency have large, especially in outpatient centers, and are not very reliable

Kruskal-Wallis test for the differences in dosage of therapy across regions yielded the following *p*-values for the inpatient individual, group, autonomous and outpatient individual, group, autonomous therapy respectively: 0.162, 0.538, 0.264, 0.077, 0.070, 0.221. Due to multiple testing the Bonferroni correction should be applied and the value 0.008 is used as threshold instead. The dosage of different types of therapies was not found as different across the regions

Individual inpatient therapy in Europe was typically reported to last for four weeks, with approximately five 45 min PT sessions being provided each week. Whilst these data vary among centres, the regional medians are approximately equivalent.

Group inpatient therapy in Europe was reported to be similar in both duration and frequency to individual therapy. It would appear from the findings that centres from Western regions use group therapy more commonly. The delivery of these group sessions was typically reported as being shorter in duration but twice as frequent as individual therapy.

Individual outpatient therapy in Europe was typically reported as being 12 weeks duration, with two 45-min sessions each week. For centres in the Eastern region, the length of therapeutic programs tend to be shorter (eight weeks) whilst centres in Western countries often reported providing therapy for 16 weeks, albeit for sessions of about 35 min duration.

Outpatient group therapy was not reported at all by centres in the Eastern region. The Northern and Western regions show a similar pattern of delivery for group therapy of 10 weeks with two sessions/week, each lasting for 60 min. In contrast the Southern region generally provides therapy over a longer time frame (20 weeks) with three sessions per week, each lasting 45 min.

Autonomous therapy was provided by too few centers to draw meaningful conclusions from the results.

Of the 72 respondents 44 (61 %) stated that the timing and duration of therapy is modified with disease severity. The modifications vary: whilst some centres reported that the intensity of programmes increases with increasing disability, others reported the opposite commenting that patients with mild to moderate disability may benefit from longer sessions/programmes. Some described a shift in emphasis from group to individual sessions with more severely disabled patients. The small differences observed between regions were not statistically significant (*p* = 0.516).

### Financial coverage of therapy

*Question 36 and 37: What percentage of the cost of inpatient (Question 36)/outpatient (Question 37) therapy is covered by the state?*

The survey respondents typically reported that state or health insurance companies covered most of the therapy fees (usual range 70–100 %; Europe-wide mean 91 % (median 100 %) for inpatient and 76 % (median 100 %) for outpatient care). Some centres reported very low coverage (20–50 %) even in inpatient settings. A minority reported regional or charity/foundation sources (data not shown).

## Discussion

In the literature, several studies have evaluated the accessibility and use of physiotherapy within individual countries from the perspective of people with MS [13–17]. No studies to date have investigated the organizational aspects of PT in MS, based on professional opinion, nor compared services between countries. This survey addresses this gap by being the first to systematically describe and compare organisational aspects across different countries in Europe. It is acknowledged that a limitation of this survey is that the majority of questions were focused on the organisation of inpatient and outpatient PT services; with less of an emphasis on community based PT, private PT clinics or PT departments within long term care. It is possible that the delivery of these services may have differed.

Whilst the high number of participating countries (*n* = 23) allows a comprehensive description of the situation across Europe, the relatively low number of centres answering the questionnaire within some countries means that the data should be interpreted with some caution. Nevertheless our response rate (37.3 %) does compare favourably with other online studies [18] where response rates range from 20 to 47 % (mean 33 %). Whilst we attempted to optimise our response rate by ensuring the questionnaire was short (two pages), and considered easy to use (as determined by our extensive piloting phase), a difficulty might have been the language barrier – there was only an English version of the questionnaire. Translation of the questionnaire to national languages might have helped to increase the response rate. An advantage of this on-line survey questionnaire approach is in high data quality due to validation checks (missing, implausible or incomplete answers, elimination of errors in the process of data entry and coding) [19].

There was no official list of workplaces providing PT for patients with MS in individual countries. The identification of candidate centres, together with the person responsible for completing the questionnaire at each centre, was therefore the responsibility of each country representative. These were experienced health professionals (clinicians or clinical researchers) involved in MS physiotherapy for more than 10 years. It is recognised that the identification of, and communication with, centre representatives might have been influenced by personality, professional knowledge and experience, together with the personal motivation and effort of each country and centre representatives. Moreover, the networking system in each country might have also influenced the with-in country response rate, which fluctuated markedly from 6 to 100 % (Table 1). The potential influence of the country representative may therefore have introduced some bias to the results. In future research, other sources should also be used (e.g. National Health Insurance Databases) to systematically identify all centres where people with MS undergo rehabilitation in order to ensure a balanced and representative sample. Whilst these factors should be taken into account in the interpretation of the results, nevertheless this is the first study to provide preliminary information about the organization of PT in MS across Europe.

The results of this survey suggest that European regions are generally similar in key organisational aspects of MS physiotherapy care including the: diversity in size and specialization of workplaces of these services; proportion of MS patients using outpatient compared to inpatient services; availability of individual face-to-face PT; reasons why people with MS are prescribed/referred for PT; and the dosage of inpatient intervention provided. There were, however, some organisational differences across regions, which included the: distribution of professionals within teams; teamwork’s working practice (uni/multi/inter-disciplinary approach); format of the PT sessions (individual, group, autonomous).

There is general agreement that people with complex needs benefit from specialist rehabilitation services [20]. Our results suggest that PT is offered to MS patients in a range of organisations - larger non-specialized hospitals, smaller specialist MS Centres, and specialist MS Rehabilitation Centres. Although the evidence base [21] and professional and patient organisations [21–23] demonstrate a preference for the delivery of services by specialist MS rehabilitation centres, only 38 % of the respondents of this European survey provide such specialist facilities. Recommendations are now in place for rehabilitation to be delivered by coordinated networks in which specialists in neurorehabilitation work within both hospital and community settings to support local generic rehabilitation and care support teams [20, 21].

A positive finding of our survey is that most participating centres reported using a teamwork approach. The role of teamwork in MS has been confirmed in several studies, as documented in a Cochrane review [24]; these however did not distinguish between multidisciplinary and interdisciplinary teamwork approaches. Our results suggest that an interdisciplinary model is slightly more frequently adopted than a multidisciplinary model; with different European regions using different teamwork models. An important research question for the future is the comparative effectiveness of these different approaches.

Our results also suggest differences in the distribution of professionals within teams from across participating centres. This can impact on effective inter-professional working [25]. For example team working can be influenced by the different priorities and roles of different professionals [26] or by the reluctance by some team members to voice opinions [27, 28].

Our survey found differences across countries with regard to who refers/prescribes PT to MS patients and the reasons for its referral/prescription. This is in line with the literature which highlights that, as yet, there remains no universally agreed criteria for patients’ referral for rehabilitation services [29]. Such criteria are an important area for future research. Ideally, patients should be referred for rehabilitation as early as possible [30]. Decision-making processes such as these are influenced by effectiveness, benefit, cost-effectiveness and cost-benefit considerations. Financial, personal, structural and attitudinal factors also influence this [31].

Differences in the types of PT offered by the participating centres were apparent in these survey results. The proportions were calculated with respect to centres offering inpatient/outpatient care. In some countries inpatient rehabilitation prevails, in others outpatient rehabilitation. Whilst individual face-to-face therapy is commonly used across Europe, group therapies and autonomy therapy concept are only used in some European regions. The length and intensity of individual inpatient therapy is broadly similar in different European regions. In contrast, many aspects of group inpatient therapy differ across countries (for example the frequency used, and duration of sessions); mainly being used in the Western region. This is also the case for outpatient therapies, both at an individual and group level. The biggest difference with regard to outpatient therapy is between Western and Eastern Europe: the length of an individual outpatient session is longer, the duration of the program is shorter, and group outpatient treatment does not occur in Eastern countries. The typical dosage of therapy per year, reported by the survey respondents, varies greatly, which is in accordance with the Cochrane review [32]. Contemporary knowledge/research does not yet provide evidence either as to what denotes an optimum ‘dose’ of therapy or the superiority of one therapy over another.

## Conclusion

This survey is the first to provide data about the organisational aspects of physiotherapy for people with MS across Europe. Overall, care in key organisational aspects of service provision is broadly similar across regions, although variations, such as the teamwork approach adopted, are apparent. These variations are likely to be determined by a combination of philosophical, cultural, economical and political factors. Our results support the notion that key organizational aspects should be reported in research protocols of studies evaluating the effectiveness of therapy and taken into consideration when planning an international multi-centre study.

## Abbreviations

MS: Multiple sclerosis
PT: Physical therapy
RIMS: Rehabilitation in Multiple Sclerosis European network of best practice and research in MS rehabilitation
